# Assessing the approaches to nausea and vomiting in pregnancy: insights from a nationwide survey of Italian gynecologists (PURITY light)

**DOI:** 10.3389/fmed.2025.1462860

**Published:** 2025-01-27

**Authors:** Romolo Di Iorio, Paola Bianchi, Elena Casolati, Elena Piccolo, Mario Mangrella

**Affiliations:** ^1^Department of Surgical and Medical Sciences and Translational Medicine, Sapienza University, Rome, Italy; ^2^Private Practice of Obstetrics and Gynecology, Milan, Italy; ^3^Department of Medical Affair, Italfarmaco SpA, Milan, Italy

**Keywords:** nausea, vomiting, pregnancy, gynecologists, pharmacotherapy, hyperemesis gravidarum

## Abstract

**Introduction:**

Nausea and vomiting in pregnancy (NVP) affect approximately 70–85% of pregnant women, starting between weeks 6 and 8 and often subsiding by week 20. This study evaluates the therapeutic approaches of Italian gynecologists in the treatment of NVP. In the Italian healthcare system, gynecologists play a central role in prenatal care and are the primary healthcare providers for pregnant women, unlike in other countries where midwives may have a more prominent role.

**Methods:**

From June to September 2022, a survey of Italian gynecologists was conducted during 15 scientific conferences. The questionnaire collected demographic data and information on clinical practices to support the management of NVP. Statistical analysis assessed the effect of demographic characteristics on clinical behavior.

**Results:**

Data from 157 participants, mainly women (72.61%) and professionals from the Public Health System (63.69%), were analyzed. 77.71% always checked NVP during the first visit, based on patients’ reports (84.71%). 54.78% prescribed medication in mild cases, mainly the doxylamine/pyridoxine combination (64.97%).

**Discussion:**

The results show varied practices influenced by demographic and professional factors. Many physicians treat only severe cases of NVP, perceiving it as a transient discomfort, which may lead to poor management. Balanced care is needed to improve the quality of life for pregnant women with NVP.

## 1 Introduction

Nausea and vomiting in pregnancy (NVP) is estimated to affect approximately 70–85% of pregnant women globally. It typically begins in the early stages of pregnancy, around the 6^th^ to 8^th^ week, and often subsides by the 16^th^ to 20^th^ week. However, for about 20% of women, symptoms may persist throughout pregnancy. Recurrence of NVP in subsequent pregnancies is also common, with rates ranging from 24 to 80%, depending on various factors such as genetics and previous pregnancy experiences (1, 2).

The proper assessment for NVP by gynecologists is essential for indicating its presence and severity, as it can significantly impact maternal and fetal health (3, 4). In the Italian healthcare system, the gynecologist plays a pivotal role in safeguarding women’s reproductive health. Specializing in the diagnosis and treatment of conditions affecting the female reproductive system, gynecologists also collaborate with other specialists to provide comprehensive care. Moreover, they support women through pregnancy, ensuring maternal and fetal well-being. Regular annual check-ups with a gynecologist are recommended for women after reaching sexual maturity, even in the absence of specific symptoms, to maintain optimal reproductive health. Currently in Italy, there are no specific national guidelines exclusively dedicated to the treatment of nausea and vomiting of pregnancy (NVP) or hyperemesis gravidarum (HG).

The Pregnancy-Unique Quantification of Emesis (PUQE) Questionnaire is a specific and validated tool designed for this purpose. The PUQE questionnaire evaluates the frequency and severity of nausea, vomiting, and retching over a specific period, providing a comprehensive assessment of NVP. PUQE questionnaire has been shown to provide strong reliability and usability in clinical and research settings (5, 6).

To effectively manage symptoms of NVP, prompt diagnosis, and treatment are crucial for improving the quality of life for pregnant women and preventing complications such as hyperemesis gravidarum (HG). Early intervention aims to alleviate the distress associated with NVP, ensuring that women maintain adequate nutritional intake and hydration, which is vital for maternal and fetal health. Effective management of NVP involves a combination of dietary modifications, lifestyle changes, and pharmacotherapy (7). The primary objectives of early diagnosis and treatment are to enhance the overall wellbeing of pregnant women by reducing the physical and psychological burdens associated with NVP symptoms. In some cases, NVP can escalate to HG, posing significant risks such as severe dehydration, electrolyte imbalances, and weight loss, potentially leading to hospitalization (8).

Additionally, there is a critical need to balance the efficacy of treatment with the safety profiles of medications used during pregnancy. Medications like the combination of doxylamine-pyridoxine have been extensively studied and typically offer effective symptom relief with a favorable safety profile. This requires a thorough assessment of any pharmacological interventions’ benefits and potential side effects (7, 8).

Various pharmacological treatments are available to manage NVP, each with different efficacy and safety profiles. Treatment practices for NVP vary significantly, influenced by both clinical guidelines and individual doctors’ attitudes. The American College of Obstetricians and Gynecologists (ACOG) recommends a combination of dietary adjustments, lifestyle changes, and pharmacotherapy tailored to the severity of symptoms and patient needs (9). However, there is often a gap between guideline recommendations and clinical practice, with some physicians hesitating to prescribe medications due to concerns about potential teratogenic effects (10, 11). At the first level, antiemetics, vitamins, and rehydration regimens can be effective for NVP (12, 13). Evidence from a multicenter trial involving 256 women showed that the combination doxylamine 10 mg/pyridoxine 10 mg resulted in a significant improvement in PUQE score and wellbeing score compared to placebo, supporting its use in treating NVP despite limited high-quality evidence on the superiority of delayed release (13). Current treatment for NVP involves several effective and safe medications. Among these, the combination of doxylamine succinate 10 mg/pyridoxine hydrochloride 10 mg is practical and safe. Since its introduction in Italy in 2018, it has been widely prescribed. However, despite the availability of effective treatments, the high psychological burden and the major impact on daily life of NVP symptoms, many women reported a lack of support from healthcare professionals and suboptimal management (14). An observational, single center prospective cohort study using validated survey instruments in pregnant women at 9–16 weeks gestation showed a low treatment utilization, even in women with moderate/severe symptoms and that gynecologists and health care providers tend to treat only severe cases of NVP, while women tend not to ask for any treatment (15). In this context, it is important to ensure proper management of nausea and vomiting in pregnancy (NVP), not only for moderate to severe cases but also for those classified as mild (4). This approach may result in a poor management of the condition, which could negatively impact the quality of life of pregnant women (16, 17).

Despite the significant impact of NVP on maternal health and quality of life, there is a notable gap in research regarding healthcare providers’ attitudes toward its management. Existing studies predominantly focus on the perspectives of General Physicians (GPs), leaving a paucity of data on the therapeutic approaches adopted by Gynecologists, who are the specialists primarily responsible for managing pregnancy-related conditions in many countries.

Other research has examined the attitudes of GPs toward the treatment of NVP, often revealing a tendency to treat only severe cases, thus potentially underestimating the condition’s broader impact (18, 19). For instance, GPs might perceive NVP as a transient and physiological discomfort of pregnancy rather than a condition necessitating proactive management. This perspective can lead to inadequate treatment and support for affected women. However, there is limited evidence on whether Gynecologists share this viewpoint or adopt different strategies for managing NVP. The role of gynecologists in prenatal care varies significantly across countries depending on the healthcare system. Understanding their attitudes and practices is essential for developing targeted interventions that ensure comprehensive care for pregnant women experiencing NVP.

Therefore, this study aimed to evaluate whether demographic and professional characteristics of gynecologists, such as age, gender, years of experience, and practice setting, are associated with specific clinical behaviors or methods for quantifying NVP. These factors were chosen based on their potential influence on decision-making and management practices.

## 2 Materials and methods

### 2.1 Data collection

From June to September 2022, a survey was conducted among Italian Gynecologists attending 15 scientific conferences. The survey was administered in paper format (Supplementary Figure 1). It was distributed during various gynecology conferences by an external provider. Upon entering the conference, physicians were asked whether they were familiar with nausea and vomiting of pregnancy (NVP) and, if so, were invited to voluntarily complete the survey without obligation. The questionnaire was anonymous and collected demographic data (gender, age, years of clinical experience, Italian geographical region, and type of employment—public or private practice) and information about the evaluation, quantification, management and treatment of pregnant patients affected by NVP.

### 2.2 Endpoints and outcomes

The questions “What is your behavior in case of NVP?” and “How do you quantify NVP?” were considered primary outcomes for NPV clinical behavior and NPV quantification, respectively.

### 2.3 Statistical analysis

For descriptive analyses, continuous variables were given as the mean with standard deviations (SD), and categorical variables were expressed as the number of subjects (n) and percentage values. The age and years of activity were categorized using 45, 55, 65, and 10, 20, 30, and 40 years as cut-offs, respectively.

A univariate analysis was performed using the multinomial log-linear regression models to assay the effect of the demographic-clinical characteristics on the NPV clinical behavior and NPV quantification. Those covariates with a p-value < 0.05 were then selected for the multivariate analysis, where the NPV clinical behavior and NPV quantification were the dependent variables. Multivariate analysis was performed using again the multinomial log-linear regression. The model selection was done using the Akaike information criterion, and the likelihood ratio test was used to measure statistical significance. The odds ratios (ORs) associated with each outcome were calculated with 95% confidence intervals (CI) for each factor by Firth’s Penalized logistic model, and all models were corrected for geographical origin.

Differences with a p-value less than 0.05 were selected as significant, and the p-values were not adjusted for multiple comparisons. Data were acquired and analyzed in the R v4.3.3 software environment (20).

## 3 Results

### 3.1 Demographic and clinical characteristics of study participants (*n* = 157)

The demographic and clinical characteristics of the study participants are summarized in Table 1. The study analyzed the demographic and clinical practice characteristics of 157 gynecologists in managing nausea and vomiting during pregnancy (NVP). Participants were predominantly female (72.61%) and working in the Public Health System (63.69%). Age distribution was varied, with 38.22% aged 56–65 years. Professional experience showed 22.29% had 21–30 years of activity, while 15.29% had ≤ 10 years. Geographically, most participants were from the center (52.87%).

**TABLE 1:** Demographic and clinical characteristics of study participants (*n* = 157).

Characteristic	Overall
**Age (years)**	
≤45	42 (26.75%)
46–55	28 (17.83%)
56–65	60 (38.22%)
>65	22 (14.01%)
NA	5 (3.18%)
**Gender**	
Male	41 (26.11%)
Female	114 (72.61%)
NA	2 (1.27%)
**Years of activity**	
≤10	24 (15.29%)
11–20	33 (21.02%)
21–30	35 (22.29%)
31–40	28 (17.83%)
>40	14 (8.92%)
NA	23 (14.65%)
**Geographical origin**	
North	19 (12.10%)
Centre	83 (52.87%)
South and Islands	53 (33.76%)
NA	2 (1.27%)
**Type of care provider**	
Public sector	100 (63.69%)
Freelance	57 (36.31%)
**How many visits per month?**	
>20	139 (88.54%)
≤20	14 (8.92%)
NA	4 (2.55%)
**How many patients do you see in the first trimester of the month?**	
>5	99 (63.06%)
≤5	57 (36.31%)
NA	1 (0.64%)

The results are expressed as mean with standard deviation or as number of subjects with percentage.

### 3.2 “What is your behavior in case of NVP?”

Most (88.54%) saw more than 20 patients per month, and 77.71% always checked for NVP during the first visit. NVP quantification was primarily based on patient reports (84.71%); in managing NVP, 54.78% prescribed drugs in mild cases to prevent progression to hyperemesis, while 26.75% treated only severe cases. Non-pharmacological treatments were preferred by 7.01% in mild cases and 7.01% in all cases, with 1.27% offering only dietary recommendations. Drug dosage evaluation was based on symptom severity by 42.68%, with the first-choice drug being doxylamine 10 mg/pyridoxine 10 mg (64.97%) (Table 2). Table 3 presents the descriptive statistics of demographic-clinical characteristics based on the responses to the question, “*What is your behavior in the case of NVP*?” The providers’ behaviors were categorized into five groups: prescribing drugs in mild cases, treating only severe cases, using non-pharmacological treatments in mild cases, exclusively using non-pharmacological treatments, and not prescribing any treatments.

**TABLE 2:** Participant practices for NVP management (*n* = 157).

Do you always check the presence of NVP during the first visit?	
*Yes*	122 (77.71%)
*Only if the patient tells me*	18 (11.46%)
*Sometime*	12 (7.64%)
*NA*	5 (3.18%)
**How do you quantify NVP?**	
*Patient reported symptom severity*	133 (84.71%)
*Individual gynecologist questionnaires*	16 (10.19%)
*With PUQE questionnaire*	5 (3.18%)
*NA*	3 (1.91%)
**What’s your behavior in case of NVP?**	
*I prescribe drugs also in mild cases to avoid the progression to hyperemesis*	86 (54.78%)
*I prescribe a treatment only in severe cases*	42 (26.75%)
*I prescribe non-pharmacological treatments only in mild cases*	11 (7.01%)
*I prescribe only non-pharmacological treatments in any cases*	11 (7.01%)
*I don’t prescribe any treatments/I give only dietary recommendations*	2 (1.27%)
*NA*	5 (3.18%)
**About prescribing drugs**	
*I evaluate the dosage based on the severity of symptoms*	67 (42.68%)
*I use the minimum effective dosage and then modify it according to the clinical response*	61 (38.85%)
*I immediately prescribe the maximum effective dosage*	22 (14.01%)
*NA*	7 (4.46%)
**When you prescribe drugs, which is your first choice?**	
*Doxylamine 10 mg/pyridoxine 10 mg*	102 (64.97%)
*Food supplements (ginger/vit. B)*	18 (11.46%)
*Sodium*+*potassium*+*citric acid*+*riboflavin*+*thiamine*+*pyridoxine*	4 (2.55%)
*Other*	5 (3.18%)
*NA*	28 (17.83%)

The results are expressed as mean with standard deviation or as number of subjects with percentage.

**TABLE 3:** Descriptive statistics of demographic-clinical characteristics in the levels of “What’s your behavior in case of NVP?” question.

	What’s your behavior in case of NVP?				
**Characteristic**	***I prescribe drugs also in mild cases to avoid the progression to hyperemesis***	***I prescribe treatment only in severe cases***	***I prescribe non-pharmacological treatments only in mild cases***	***I prescribe only non-pharmacological treatments in any case***	***I don’t prescribe any treatments/I give only dietary recommendations***
	***N* = 86**	***N* = 42**	***N* = 11**	***N* = 11**	***N* = 2**
**Geographical origin**					
*North*	10 (52.63%)	8 (42.11%)	1 (5.26%)	0 (0%)	0 (0%)
*Centre*	38 (47.5%)	27 (33.75%)	7 (8.75%)	7 (8.75%)	1 (1.25%)
*South and Islands*	36 (70.59%)	7 (13.73%)	3 (5.88%)	4 (7.84%)	1 (1.96%)
**Gender**					
*Male*	19 (47.5%)	17 (42.5%)	1 (2.5%)	1 (2.5%)	2 (5%)
*Female*	65 (59.09%)	25 (22.73%)	10 (9.09%)	10 (9.09%)	0 (0%)
**Age *(years)***					
*≤45*	29 (70.73%)	5 (12.2%)	5 (12.2%)	2 (4.88%)	0 (0%)
*46–55*	14 (50%)	9 (32.14%)	1 (3.57%)	4 (14.29%)	0 (0%)
*56–65*	30 (52.63%)	20 (35.09%)	3 (5.26%)	3 (5.26%)	1 (1.75%)
*>65*	9 (42.86%)	8 (38.1%)	1 (4.76%)	2 (9.52%)	1 (4.76%)
**Years of activity**					
*≤10*	20 (83.33%)	2 (8.33%)	2 (8.33%)	0 (0%)	0 (0%)
*11–20*	16 (50%)	9 (28.12%)	4 (12.5%)	3 (9.38%)	0 (0%)
*21–30*	17 (50%)	12 (35.29%)	2 (5.88%)	3 (8.82%)	0 (0%)
*31–40*	14 (51.85%)	9 (33.33%)	1 (3.7%)	1 (3.7%)	2 (7.41%)
*>40*	8 (61.54%)	4 (30.77%)	1 (7.69%)	0 (0%)	0 (0%)
**Type of care provider**					
*Public sector*	56 (56.57%)	29 (29.29%)	8 (8.08%)	6 (6.06%)	0 (0%)
*Freelance*	30 (56.6%)	13 (24.53%)	3 (5.66%)	5 (9.43%)	2 (3.77%)
**How many visits per month?**					
*>20*	79 (58.52%)	35 (25.93%)	9 (6.67%)	11 (8.15%)	1 (0.74%)
*≤20*	5 (38.46%)	5 (38.46%)	2 (15.38%)	0 (0%)	1 (7.69%)
**How many patients do you see in the first trimester of the month?**					
* > 5*	63 (64.95%)	20 (20.62%)	7 (7.22%)	7 (7.22%)	0 (0%)
* ≤ 5*	22 (40.74%)	22 (40.74%)	4 (7.41%)	4 (7.41%)	2 (3.7%)
**Do you always check the presence of NVP during the first visit?**					
*Yes*	72 (60%)	30 (25%)	10 (8.33%)	7 (5.83%)	1 (0.83%)
*Only if the patient tells me*	8 (47.06%)	6 (35.29%)	1 (5.88%)	2 (11.76%)	0 (0%)
*Sometime*	6 (50%)	3 (25%)	0 (0%)	2 (16.67%)	1 (8.33%)
**How do you quantify NVP?**					
*Patient reported symptom severity*	70 (53.44%)	39 (29.77%)	9 (6.87%)	11 (8.4%)	2 (1.53%)
*Individual gynecologist questionnaires*	11 (73.33%)	3 (20%)	1 (6.67%)	0 (0%)	0 (0%)
*With PUQE questionnaire*	5 (100%)	0 (0%)	0 (0%)	0 (0%)	0 (0%)
**About prescribing drugs**					
*I evaluate the dosage based on the severity of symptoms*	37 (55.22%)	24 (35.82%)	3 (4.48%)	3 (4.48%)	0 (0%)
*I use the minimum effective dosage and then modify it according to the clinical response*	33 (54.1%)	13 (21.31%)	7 (11.48%)	6 (9.84%)	2 (3.28%)
*I immediately prescribe the maximum effective dosage*	16 (72.73%)	4 (18.18%)	1 (4.55%)	1 (4.55%)	0 (0%)
**When you prescribe drugs, which is your first choice?**					
*Doxylamine 10 mg/pyridoxine 10 mg*	70 (68.63%)	21 (20.59%)	6 (5.88%)	4 (3.92%)	1 (0.98%)
*Food supplements (ginger/vit. B)*	7 (38.89%)	4 (22.22%)	2 (11.11%)	4 (22.22%)	1 (5.56%)
*Sodium+potassium+citric acid+riboflavin+* *thiamine+pyridoxine*	2 (50%)	1 (25%)	1 (25%)	0 (0%)	0 (0%)
*Other*	1 (20%)	2 (40%)	0 (0%)	2 (40%)	0 (0%)

Notably, only two providers exclusively offered dietary recommendations or no treatments, and this category was excluded from further univariate and multivariate analysis. As concern gender, it seemed that females were different from males in terms of prescribing practices sure enough females preferred prescribing drugs in mild cases (59.09%), while males were inclined to choose both prescribing drugs in mild cases and treating only severe cases (47.50 and 42.5%, respectively). Furthermore again in terms of prescribing practices, a different trend was observed in the number of patients seen in the first quarter of the month. Particularly, the providers who had seen > 5 patients tended to prescribe drugs in mild cases (64.95%), instead who had visited five or fewer patients preferred to get both prescribing drugs in mild cases and treating only severe cases (40.74 and 40.74%, respectively).

A significant finding was that one provider seeing over 20 patients per month did not prescribe any treatment, highlighting potential variability in clinical behavior. Age influenced prescribing patterns; younger gynecologists (≤45 years) more frequently prescribed drugs for mild cases (70.73%), while older practitioners > 65 years) demonstrated a more conservative approach, prescribing non-pharmacological treatments in 9.52% of cases.

Years of clinical activity influenced practices, with 83.33% of those prescribing drugs in mild cases having ≤ 10 years of experience. Providers not prescribing treatments had extensive experience, with all having 31–40 years of activity. The PUQE questionnaire was underutilized, applied exclusively by 5 respondents for quantifying NVP severity, with most relying on patient-reported symptoms (53.44%). Doxylamine-pyridoxine was the first choice for drug therapy among 68.63% of prescribers, highlighting its preference across severity levels.

A univariate analysis (Supplementary Table 1), corrected for geographical origin, was conducted on the responses to the question “*What is your behavior in case of NVP*?” among 150 participants. The results revealed several interesting findings such as gender, that it was significantly impacted on prescribing behaviors (*p* = 0.0171), with female providers less likely than males to prescribe treatment only in severe cases (OR = 0.38, 95% CI: 0.16:0.87). Although not statistically significant, females were more inclined to prescribe non-pharmacological treatments in mild cases (OR = 2.08, 95% CI: 0.44:20.11) and in many cases (OR = 2.06, 95% CI: 0.44:19.98). Age showed notable trends, though not statistically significant overall (*p* = 0.1011). Providers aged 46–55 had higher odds of prescribing treatment only in severe cases (OR = 3.78, 95% CI: 1.1:14.22) compared to those ≤ 45 years old, with even higher odds for those aged 56–65 (OR = 4.08, 95% CI: 1.43:13.35) and > 65 years (OR = 4.47, 95% CI: 1.22:17.88). Years of activity slightly influence clinical practice (*p* = 0.1448), with providers having 11–20 years of practice showing higher odds (OR = 4.33, 95% CI: 1.01–25.64), and those with 21–30 years (OR = 6.82, 95% CI: 1.64:40.2) and 31–40 years (OR = 6.05, 95% CI: 1.39:36.55) even more likely to prescribe in severe cases. The type of care provider (public vs. freelance) did not significantly affect prescribing behaviors (*p* = 0.6090). However, the number of patients seen in the first trimester was significant (*p* = 0.0313), with providers seeing ≤ 5 patients more likely to prescribe the treatment only in severe cases (OR = 3.37, 95% CI: 1.51:7.77). Checking for NVP during the first visit did not significantly influence treatment choices (*p* = 0.5597). However, providers who checked only if the patient mentioned symptoms had higher odds of prescribing non-pharmacological treatments in any case (OR = 3.25, 95% CI: 0.53:16.02). The method of quantifying NVP showed no significant impact (*p* = 0.2121), with most providers relying on patient reports. Evaluating drug dosage based on symptom severity was the most common approach. However, using the minimum effective dosage or immediately prescribing the maximum effective dosage did not significantly influence treatment decisions (*p* = 0.1974). The choice of first-line treatment showed some notable trends (*p* = 0.0686), with providers who chose food supplements (ginger/vit. B) being more likely to prescribe non-pharmacological treatments in any case (OR = 7.55, 95% CI: 1.66:35.31), and those selecting “Other” treatments had significantly higher odds for this behavior (OR = 19.96, 95% CI: 2.23:252.8).

The subsequent multivariate analysis of responses to the question “*What is your behavior in case of NVP?*” (*N* = 146), corrected for geographical origin, provides insightful findings on prescribing practices and a result summary are report in Table 4. Geographical origin significantly influenced prescribing behaviors (*p* = 0.0044). Compared to the North, both providers in the Centre and South & Islands were less likely to prescribe treatment only in severe cases (OR = 0.67, 95% CI: 0.22:2.05 and 0.17 (0.04:0.62), respectively). Gender also played a significant role in treatment decisions (*p* = 0.0159). Taking for reference the male gender, female providers were 65% less likely in prescribing treatment only in severe cases (OR = 0.35, 95% CI: 0.14:0.83). They were more likely to prescribe non-pharmacological treatments only in mild cases (OR = 1.98, 95% CI: 0.42:19.11) and non-pharmacological treatments in any case (OR = 2.04, 95% CI: 0.41:20.34). However, these latter associations were not statistically significant. The number of patients seen in the first trimester of pregnancy significantly affected prescribing behavior (*p* = 0.0337). Providers seeing five or fewer patients were significantly about 3.5 times more likely to prescribe treatment only in severe cases (OR = 3.52, 95% CI: 1.54:8.38). These providers were more likely to prescribe non-pharmacological treatments only in mild cases (OR = 1.47, 95% CI: 0.37:5.25) and non-pharmacological treatments in any case (OR = 1.15, 95% CI: 0.29:4.15). However, these latter associations did not reach statistical significance.

**TABLE 4:** Multivariate analysis, corrected for geographical origin, on the question “What’s your behavior in case of NVP?” (*N* = 146).

Characteristic	*I prescribe a treatment only in severe cases* vs. *I prescribe drugs also in mild cases to avoid the progression to hyperemesis*	*I prescribe non-pharmacological treatments only in mild cases* vs. *I prescribe drugs also in mild cases to avoid the progression to hyperemesis*	*I prescribe only non-pharmacological treatments in any cases* vs. *I prescribe drugs also in mild cases to avoid the progression to hyperemesis*	*p*-value
**Geographical origin**				0.0044
*North*	1	1	1	
*Centre*	0.67 (0.22:2.05)	1.31 (0.23:13.72)	4.04 (0.41:547.72)	
*South and Islands*	0.17 (0.04:0.62)	0.65 (0.09:7.49)	2.73 (0.25:377.09)	
**Gender**				0.0159
*Male*	1	1	1	
*Female*	0.35 (0.14:0.83)	1.98 (0.42:19.11)	2.04 (0.41:20.34)	
**How many patients do you see in the first trimester of the month?**				0.0337
*>5*	1	1	1	
*≤5*	3.52 (1.54:8.38)	1.47 (0.37:5.25)	1.15 (0.29:4.15)	

Results are expressed as odds ratio (OR) with 95% confidence interval (95%CI); *p*-value: Likelihood Ratio *p*-value.

### 3.3 “How do you quantify NVP?”

The demographic and clinical characteristics based on the methods used to quantify NVP are summarized in Table 5. Briefly, all demographic and clinical subgroups of patients had similar distribution in responded percentages to the question, “How do you quantify NVP?” The most frequent quantification of NPV in all subgroups of patients was by patient reported symptom severity. The univariate analysis corrected for geographical origin in Supplementary Table 2 explores factors influencing healthcare providers’ methods for quantifying NVP. Gender did not significantly impact the choice of quantification method (*p* = 0.3151), though female providers were somewhat more likely to use personal questionnaires (OR = 2.42, 95% CI: 0.68–12.90) and the PUQE questionnaire (OR = 1.31, 95% CI: 0.23–13.55). Age did not show a significant effect (*p* = 0.5227), with higher odds for personal questionnaire use in the 46–55 age group (OR = 3.11, 95% CI: 0.64–18.99) and both methods in the 56–65 age group (personal OR = 2.66, 95% CI: 0.68–14.67; PUQE OR = 1.71, 95% CI: 0.27–18.16). Years of activity showed a light trend (*p* = 0.2620), particularly for those with over 40 years of experience, who had higher odds of using personal (OR = 14.15, 95% CI: 1.02–2031.85) and PUQE (OR = 6.71, 95% CI: 0.32–1028.8) questionnaires. The type of care provider (public vs. private) was not a significant factor (*p* = 0.8484). Neither the number of visits per month (*p* = 0.6400) nor the number of patients seen in the first trimester (*p* = 0.5601) significantly affected the choice of method. Providers who checked for NVP only if mentioned by the patient were more likely to use the PUQE questionnaire (OR = 2.08, 95% CI: 0.19–12.91). Prescribing behavior in cases of NVP did not show significant differences (*p* = 0.3401), with those prescribing drugs in mild cases being the reference group. Regarding drug prescription practices (*p* = 0.5311), providers using the minimum effective dosage had higher odds of using personal (OR = 1.64, 95% CI: 0.53–5.45) and PUQE (OR = 1.74, 95% CI: 0.22–19.47) questionnaires, while those prescribing the maximum effective dosage had higher odds of using the PUQE method (OR = 5.66, 95% CI: 0.70–66.22). The preference for doxylamine 10 mg/pyridoxine 10 mg as the first-choice drug did not significantly impact the quantification method (*p* = 0.7227).

**TABLE 5:** Descriptive statistics of demographic-clinical characteristics in the levels of “How do you quantify NVP?” question.

Characteristic	How do you quantify NVP?		
	***Patient reported symptom severity***	***Individual gynecologist questionnaires***	***With PUQE questionnaire***
**Geographical origin**			
*North*	17 (89.47%)	1 (5.26%)	1 (5.26%)
*Centre*	72 (88.89%)	8 (9.88%)	1 (1.23%)
*South & Islands*	42 (80.77%)	7 (13.46%)	3 (5.77%)
**Gender**			
*Male*	37 (92.5%)	2 (5%)	1 (2.5%)
*Female*	95 (84.82%)	13 (11.61%)	4 (3.57%)
**Age *(years)***			
*=45*	39 (92.86%)	2 (4.76%)	1 (2.38%)
*46–55*	24 (85.71%)	4 (14.29%)	0 (0%)
*56–65*	48 (81.36%)	8 (13.56%)	3 (5.08%)
*>65*	18 (85.71%)	2 (9.52%)	1 (4.76%)
**Years of activity**			
*≤10*	24 (100%)	0 (0%)	0 (0%)
*11–20*	29 (87.88%)	3 (9.09%)	1 (3.03%)
*21–30*	29 (82.86%)	5 (14.29%)	1 (2.86%)
*31–40*	24 (85.71%)	2 (7.14%)	2 (7.14%)
*>40*	9 (75%)	2 (16.67%)	1 (8.33%)
**Type of care provider**			
*Public sector*	85 (85.86%)	10 (10.1%)	4 (4.04%)
*Freelance*	48 (87.27%)	6 (10.91%)	1 (1.82%)
**How many visits per month?**			
*>20*	120 (86.96%)	14 (10.14%)	4 (2.9%)
*≤20*	10 (83.33%)	2 (16.67%)	0 (0%)
**How many patients do you see in the first trimester of the month?**			
*>5*	84 (84.85%)	12 (12.12%)	3 (3.03%)
*≤5*	48 (88.89%)	4 (7.41%)	2 (3.7%)
**Do you always check the presence of NVP during the first visit?**			
*Yes*	104 (85.95%)	13 (10.74%)	4 (3.31%)
*Only if the patient tells me*	16 (88.89%)	1 (5.56%)	1 (5.56%)
*Sometime*	10 (83.33%)	2 (16.67%)	0 (0%)
**What’s your behavior in case of NVP?**			
*I prescribe drugs also in mild cases to avoid the progression to hyperemesis*	70 (81.4%)	11 (12.79%)	5 (5.81%)
*I prescribe treatment only in severe cases*	39 (92.86%)	3 (7.14%)	0 (0%)
*I prescribe non-pharmacological treatments only in mild cases*	9 (90%)	1 (10%)	0 (0%)
*I prescribe only non-pharmacological treatments in any cases*	11 (100%)	0 (0%)	0 (0%)
*I don’t prescribe any treatments/I give only dietary recommendations*	2 (100%)	0 (0%)	0 (0%)
**About prescribing drugs**			
*I evaluate the dosage based on the severity of symptoms*	60 (90.91%)	5 (7.58%)	1 (1.52%)
*I use the minimum effective dosage and then modify it according to the clinical response*	51 (83.61%)	8 (13.11%)	2 (3.28%)
*I immediately prescribe the maximum effective dosage*	18 (81.82%)	2 (9.09%)	2 (9.09%)
**When you prescribe drugs, which is your first choice?**			
*Doxylamine 10 mg/pyridoxine 10 mg*	85 (83.33%)	13 (12.75%)	4 (3.92%)
*Food supplements (ginger/vit. B)*	16 (88.89%)	1 (5.56%)	1 (5.56%)
*Sodium+potassium+citric acid+riboflavin+thiamine+pyridoxine*	3 (100%)	0 (0%)	0 (0%)
*Other*	5 (100%)	0 (0%)	0 (0%)

## 4 Discussion

The study provides an analysis of the management of NVP among 157 healthcare providers in Italy, highlighting significant variability in clinical practices.

A notable finding is that 88.54% of gynecologists see more than 20 patients per month, yet only 77.71% consistently check NVP during the first visit. This suggests a critical gap in routine screening practices, given that early identification and management of NVP can prevent progression to more severe forms like hyperemesis gravidarum.

In this study, the use of the Pregnancy-Unique Quantification of Emesis (PUQE) questionnaire was limited, with only 3.18% of providers utilizing it. This underscores the need for standardized assessment tools in clinical practice to ensure consistent and accurate diagnosis and treatment, as reported in the literature (13).

In this study, treatment practices vary significantly, with 54.78% of providers prescribing drugs even in mild cases of NVP to prevent progression, while 26.75% only prescribe treatment in severe cases. This discrepancy highlights differing clinical approaches and potential under-treatment of NVP in its milder forms, which can negatively impact maternal health and quality of life (21).

This study’s preference for doxylamine/pyridoxine as the first-choice medication (64.97%) aligns with existing guidelines that recommend this combination due to its established efficacy and safety profile (13).

Geographical and gender differences also influence treatment practices. Providers from the Centre, South, and Islands are less likely to prescribe drugs in mild cases compared to those from the North, indicating regional variations in clinical management. Female providers are generally less likely to treat only severe cases than their male counterparts, possibly reflecting a greater sensitivity to the burdens of NVP (22, 23).

The multivariate analysis further supports these findings, showing that gender, geographical location, and the number of patients seen influence treatment decisions. Female providers and those seeing fewer patients in the first trimester are more likely to prescribe treatment only in severe cases, suggesting that workload and provider characteristics significantly impact clinical practices (23).

This study presents some limitations. Firstly, the sample size of 157 gynecologists may not represent the entire population of gynecologists in Italy, affecting the generalizability of the findings.

Also, the survey did not provide a standard definition of mild versus severe NVP to the study participants, which may have introduced variability in the responses. While the PUQE (Pregnancy-Unique Quantification of Emesis) scoring system is a commonly used tool for categorizing NVP severity, it was not explicitly applied in this study. Consequently, participants’ interpretations of mild and severe NVP may have varied, reflecting their individual clinical practices and experiences. This variability should be considered a limitation, as it may have influenced the consistency and comparability of the reported management practices.

In conclusion, the study highlights significant variability in the management of nausea and vomiting in pregnancy (NVP) among Italian gynecologists, influenced by demographic factors, regional differences, and provider characteristics, indicating the need for standardized guidelines and consistent screening practices to ensure comprehensive care and prevent progression to more severe conditions like hyperemesis gravidarum.

Future research should prioritize identifying optimal screening and treatment strategies for NVP and developing targeted educational initiatives to enhance clinicians’ adherence to best practices, ultimately improving patient outcomes (21, 24).

The goal of this study is to raise awareness among physicians about NVP and the use of pharmacological treatment not only in severe cases. The study highlights significant variability in the management of NVP among Italian gynecologists, influenced by demographic and professional factors. To date only the Purity study has evaluated the prevalence and treatment of NVP in Italy; with this additional survey we aimed to have additional data that could be of interest to develop national guidelines on NVP that currently do not exist. The findings underscore the need for standardized assessment tools and more consistent screening and management strategies to ensure effective and comprehensive care for pregnant women experiencing NVP.

## Data Availability

The original contributions presented in the study are included in the article/Supplementary material, further inquiries can be directed to the corresponding author.
