# Supplementary material for: Assessing the approaches to nausea and vomiting in pregnancy: insights from a nationwide survey of Italian gynecologists (PURITY light)
Source: Front Med (Lausanne). 2025 Jan 27;12:1462860. doi: 10.3389/fmed.2025.1462860 (PMC11808362; doi:10.3389/fmed.2025.1462860)
Supplement: Supplementary file 1 [file Supplementary_file_1.docx]

**Supplementary materials**

**Figure S1:** Questions and answers included in the survey provided to gynecologists.


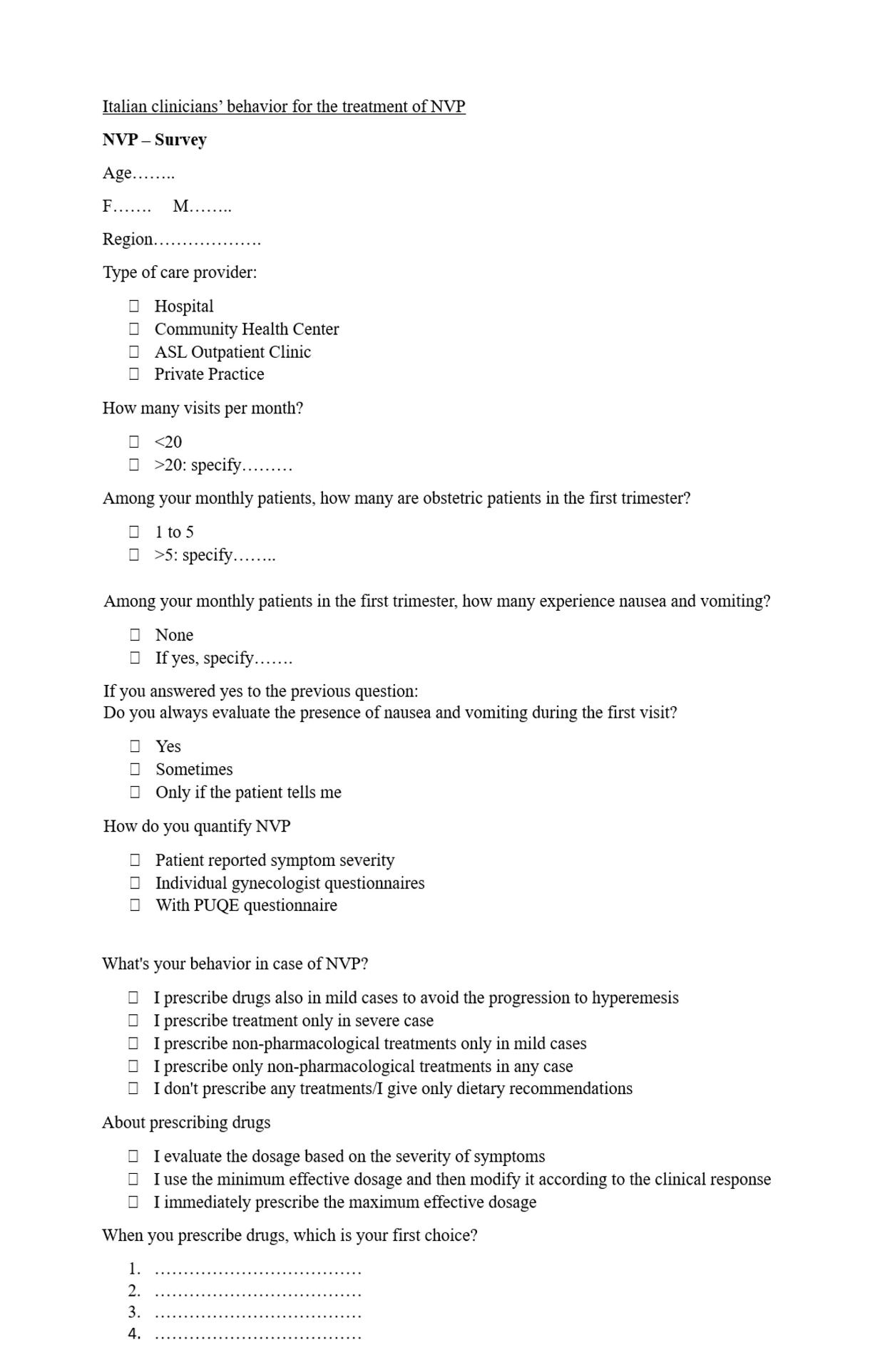


**Table S1**: Univariate analysis, corrected for geographical origin, on the question “What's your behavior in case of NVP?” (N=150).

Results are expressed as odds ratio (OR) with 95% confidence interval (95%CI); p-value: Likelihood Ratio p-value.

| **Characteristic** | ***I prescribe treatment only in severe cases*** | ***I prescribe non-pharmacological treatments only in mild cases*** | ***I prescribe only non-pharmacological treatments in any case*** | **p-value** |
| --- | --- | --- | --- | --- |
|  | *versus* | *versus* | *versus* |  |
|  | ***I prescribe drugs also in mild cases to avoid the progression to hyperemesis*** | ***I prescribe drugs also in mild cases to avoid the progression to hyperemesis*** | ***I prescribe drugs also in mild cases to avoid the progression to hyperemesis*** |  |
| **Gender** |  |  |  | 0.0171 |
| *Male* | 1 | 1 | 1 |  |
| *Female* | 0.38 (0.16 : 0.87) | 2.08 (0.44 : 20.11) | 2.06 (0.44 : 19.98) |  |
| **Age** *(years)* |  |  |  | 0.1011 |
| *<=45* | 1 | 1 | 1 |  |
| *46-55* | 3.78 (1.1 : 14.22) | 0.66 (0.06 : 3.74) | 4.44 (0.85 : 28.76) |  |
| *56-65* | 4.08 (1.43 : 13.35) | 0.65 (0.14 : 2.64) | 1.46 (0.26 : 9.37) |  |
| *>65* | 4.47 (1.22 : 17.88) | 0.84 (0.08 : 4.98) | 3.18 (0.43 : 23.74) |  |
| **Years of activity** |  |  |  | 0.1448 |
| *≤ 10* | 1 | 1 | 1 |  |
| *11-20* | 4.33 (1.01 : 25.64) | 2.23 (0.44 : 14.11) | 9.58 (0.83 : 1328.35) |  |
| *21-30* | 6.82 (1.64 : 40.2) | 1.29 (0.18 : 9.34) | 8.43 (0.73 : 1169.46) |  |
| *31-40* | 6.05 (1.39 : 36.55) | 0.81 (0.07 : 6.80) | 3.87 (0.19 : 580.05) |  |
| *>40* | 3.99 (0.71 : 27.73) | 1.49 (0.12 : 12.97) | 2.85 (0.01 : 548.63) |  |
| **Type of care provider** |  |  |  | 0.6090 |
| *Public sector* | 1 | 1 | 1 |  |
| *Freelance* | 0.64 (0.28 : 1.45) | 0.67 (0.15 : 2.40) | 1.45 (0.39 : 5.27) |  |
| **How many visits per month?** |  |  |  | 0.1467 |
| *>20* | 1 | 1 | 1 |  |
| *≤ 20* | 2.63 (0.69 : 10.17) | 4.02 (0.63 : 21.11) | 0.57 (0 : 5.62) |  |
| **How many patients do you see in the first trimester of the month?** |  |  |  | 0.0313 |
| *>5* | 1 | 1 | 1 |  |
| *≤ 5* | 3.37 (1.51 : 7.77) | 1.63 (0.42 : 5.74) | 1.41 (0.36 : 4.89) |  |
| **Do you always check the presence of NVP during the first visit?** |  |  |  | 0.5597 |
| *Yes* | 1 | 1 | 1 |  |
| *Only if the patient tells me* | 1.51 (0.47 : 4.71) | 1.29 (0.13 : 6.83) | 3.25 (0.53 : 16.02) |  |
| *Sometime* | 1.57 (0.34 : 6.48) | 0.62 (0.01 : 6.66) | 3.88 (0.60 : 21.16) |  |
| **How do you quantify NVP?** |  |  |  | 0.2121 |
| *Patient reported symptom severity* | 1 | 1 | 1 |  |
| *Individual gynecologist questionnaires* | 0.58 (0.14 : 1.96) | 1.02 (0.10 : 5.22) | 0.27 (0.01 : 2.35) |  |
| *With PUQE questionnaire* | 0.19 (0.01 : 1.90) | 0.74 (0.01 : 7.60) | 0.65 (0.01 : 6.8) |  |
| **About prescribing drugs** |  |  |  | 0.1974 |
| *I evaluate the dosage based on the severity of symptoms* | 1 | 1 | 1 |  |
| *I use the minimum effective dosage and then modify it according to the clinical response* | 0.62 (0.26 : 1.43) | 2.3 (0.62 : 10.23) | 2.22 (0.57 : 10.10) |  |
| *I immediately prescribe the maximum effective dosage* | 0.39 (0.11 : 1.22) | 0.98 (0.09 : 6.59) | 1.10 (0.10 : 7.50) |  |
| **When you prescribe drugs, which is your first choice?** |  |  |  | 0.0686 |
| *doxylamine 10mg/pyridoxine 10mg* | 1 | 1 | 1 |  |
| *food supplements (ginger/vit. B)* | 2.35 (0.58 : 9.15) | 3.17 (0.51 : 15.73) | 7.55 (1.66 : 35.31) |  |
| *sodium+potassium+citric acid+riboflavin+thiamine+ pyridoxine* | 2.03 (0.16 : 18.77) | 4.96 (0.40 : 43.9) | 2.57 (0.02 : 38.16) |  |
| *Other* | 5.78 (0.61 : 81.56) | 4.16 (0.03 : 100.11) | 19.96 (2.23 : 252.8) |  |

**Table S2**: Univariate analysis, corrected for geographical origin, on the question “How do you quantify NVP?” (N=157).

Results are expressed as odds ratio (OR) with 95% confidence interval (95%CI); p-value: Likelihood Ratio p-value.

| **Characteristic** | ***Individual gynecologist questionnaires*** | ***With PUQE questionnaire*** | **p-value** |
| --- | --- | --- | --- |
|  | *versus* | *versus* |  |
|  | ***Patient reported symptom severity*** | ***Patient reported symptom severity*** |  |
| **Gender** |  |  | 0.3151 |
| *Male* | 1 | 1 |  |
| *Female* | 2.42 (0.68 : 12.90) | 1.31 (0.23 : 13.55) |  |
| **Age** *(years)* |  |  | 0.5227 |
| *<=45* | 1 | 1 |  |
| *46-55* | 3.11 (0.64 : 18.99) | 0.59 (0.01 : 11.61) |  |
| *56-65* | 2.66 (0.68 : 14.67) | 1.71 (0.27 : 18.16) |  |
| *>65* | 2.19 (0.32 : 15.19) | 1.99 (0.15 : 25.81) |  |
| **Years of activity** |  |  | 0.2620 |
| *≤ 10* | 1 | 1 |  |
| *11-20* | 6 (0.54 : 821.95) | 2.59 (0.13 : 385.62) |  |
| *21-30* | 8.93 (0.94 : 1192.73) | 2.25 (0.11 : 334.97) |  |
| *31-40* | 4.72 (0.36 : 664.2) | 4.89 (0.37 : 689.24) |  |
| *>40* | 14.15 (1.02 : 2031.85) | 6.71 (0.32 : 1028.8) |  |
| **Type of care provider** |  |  | 0.8484 |
| *Public sector* | 1 | 1 |  |
| *Freelance* | 1.16 (0.39 : 3.28) | 0.74 (0.07 : 4.24) |  |
| **How many visits per month?** |  |  | 0.6400 |
| *>20* | 1 | 1 |  |
| *≤ 20* | 1.72 (0.31 : 6.82) | 1.03 (0.01 : 12.38) |  |
| **How many patients do you see in the first trimester of the month?** |  |  | 0.5601 |
| *>5* | 1 | 1 |  |
| *≤ 5* | 0.59 (0.17 : 1.74) | 1.23 (0.20 : 6.73) |  |
| **Do you always check the presence of NVP during the first visit?** |  |  | 0.7482 |
| *Yes* | 1 | 1 |  |
| *Only if the patient tells me* | 0.71 (0.07 : 3.24) | 2.08 (0.19 : 12.91) |  |
| *Sometime* | 1.61 (0.28 : 6.53) | 0.86 (0.01 : 9.18) |  |
| **What's your behavior in case of NVP?** |  |  | 0.3401 |
| *I prescribe drugs also in mild cases to avoid the progression to hyperemesis* | 1 | 1 |  |
| *I prescribe treatment only in severe cases* | 0.61 (0.15 : 2.06) | 0.18 (0.01 : 1.76) |  |
| *I prescribe non-pharmacological treatments only in mild cases* | 0.98 (0.10 : 4.95) | 0.77 (0.01 : 8.10) |  |
| *I prescribe only non-pharmacological treatments in any cases* | 0.25 (0.01 : 2.2) | 0.64 (0.01 : 6.80) |  |
| *I don't prescribe any treatments/I give only dietary recommendations* | 1.08 (0.01 : 14.52) | 2.45 (0.02 : 39.98) |  |
| **About prescribing drugs** |  |  | 0.5311 |
| *I evaluate the dosage based on the severity of symptoms* | 1 | 1 |  |
| *I use the minimum effective dosage and then modify it according to the clinical response* | 1.64 (0.53 : 5.45) | 1.74 (0.22 : 19.47) |  |
| *I immediately prescribe the maximum effective dosage* | 1.47 (0.25 : 6.69) | 5.66 (0.70 : 66.22) |  |
| **When you prescribe drugs, which is your first choice?** |  |  | 0.7227 |
| *doxylamine 10mg/pyridoxine 10mg* | 1 | 1 |  |
| *food supplements (ginger/vit. B)* | 0.53 (0.06 : 2.44) | 1.57 (0.15 : 9.53) |  |
| *sodium+potassium+citric acid+riboflavin+thiamine+ pyridoxine* | 0.84 (0.01 : 9.59) | 2.92 (0.02 : 44.35) |  |
| *Other* | 0.62 (0.01 : 6.04) | 1.73 (0.01 : 20.84) |  |
